# Seasonal histological changes and aquaporin 5 immunoreactivity in the ductus deferens and prostate gland of Saudi Arabian dromedary camel

**DOI:** 10.5455/javar.2024.k820

**Published:** 2024-09-30

**Authors:** Marwa Babiker

**Affiliations:** 1 Department of Anatomy, College of Veterinary Medicine, King Faisal University, Al-Ahsa, Saudi Arabia; 2 Department of Anatomy, College of Veterinary Medicine, University of Bahri, Khartoum North, Sudan

**Keywords:** Seasonal histology, camel, ductus deferens, prostate

## Abstract

**Objectives:**

The present study aims to investigate the seasonal histological structure changes and immunoreactivity of Aquaporin 5 (AQP-5) in the ductus deferens and prostate under the light microscope.

**Materials and Methods:**

For the present study, twelve mature male camels were employed. Following the animals breeding and non-breeding seasons of slaughter under official licensing and veterinary supervision, samples were obtained from various regions of the ductus deferens and prostate gland and processed using general histology and immunohistochemical methods.

**Results:**

The secretory end pieces of the vas deferens ampulla and prostate gland of the Saudi Arabian dromedary camel were well developed during reproductive and non-reproductive seasons. The spermatozoa were noticed in the lumen of the ductus deferens during the reproductive and non-reproductive seasons. The response of AQP-5 in the ductus deferens was particularly significant throughout the not-rutting season. Moreover, during the non-breeding season, the prostate gland showed a strong to moderate response. In the breeding season, the ampulla of the ductus deferens showed an insignificant reaction. Additionally, the body and disseminated portion of the prostate gland showed a mild to moderate response.

**Conclusion:**

The results suggested that Saudi Arabian dromedary camels might produce sperm during rutting and non-rutting seasons.

## Introduction

Dromedary camels are able to survive in a variety of dry and semi-arid situations because camels are highly adapted to the habitats they live in [1,2]. Seasonality in reproduction has been observed in different mammalian species, including male camels [3]. It appears that camels have a longer breeding season than previously thought. Dromedary camels are recognized as seasonal breeders [4]. According to Marai et al. [5], the male camel breeds only at specific times of the year, and the breeding season is characterized by a notable increase in sexual activity or the rut. It is still possible for the male to fertilize the oocyte by mating with a female in estrus at any time of year. The embryonic origin of the vas deferens is from the intermediate mesoderm (mesonephric or Wolffian ducts) [6]. The primary function of the ductus deferens is to carry spermatozoa during emission from the epididymis to the ejaculatory duct. An essential function of the ductus deferens is to nourish, store, and mature spermatozoa [7,8]. The vesicular gland’s excretory duct and the ductus deferens in stallions and ruminants join to make the short ejaculatory duct that exits into the urethra at the colliculus seminalis [9].

The prostate gland is a secondary sexual organ that incorporates secretions into the seminal fluid [7,10,11]. However, the peripheral zone, the central zone, and the transition zone are the three main zones of the prostate gland, and they are each different histologically and biologically [12].

Several investigators examined the structure of the accessory glands in male camels [13–16]. Research concerning the conducted seasonal histological changes in the vas deferens and the prostate gland in the available literature is scant, for instance; those were reported previously [3,17,18].

Every living organism, including people and microorganisms, contains aquaporins (AQPs), which are necessary membrane proteins. AQPs are found in a wide range of human tissues and are involved in the bidirectional transmembrane diffusion of various small solutes and water. By enabling the quick passive movement of water, they regulate the flow of fluids in cells and tissues, including the male reproductive organs [19,20,21]. There are thirteen different isoforms of AQPs in mammals, ranging from AQP0 to AQP12 [22].

This research aims to examine the seasonal variations in histology in the ductus deferens and prostate gland of the Saudi Arabian local camel breed during reproductive and non-reproductive seasons and changes, as well as to detect AQP5 in the ductus deferens and prostate gland using histological and immunohistochemistry techniques.

## Materials and Methods

### Ethical approval

The Saudi Arabian Ministry of Environment, Water, and Agriculture’s ethical standards and protocols for animal slaughter were followed during every stage of the animal sample process. The animal sampling was approved by the King Faisal University ethics committee (NOV-ETHICS1545-KFU-REC-2023).

### Samples

Twelve (12) mature and healthy adult Saudi Arabian male camels (*Camelus dromedarius*) (age 4–8 years) were used in this study. The animals were slaughtered at the Al-Omran slaughterhouse in Al-Ahsa, Saudi Arabia. Six animals during the mating season (December-February) and six animals during the non-matting season (May-August).

### Samples processing

For histological investigation, the samples were taken from the ductus deferens (initial, middle, and ampulla parts) and prostate gland (body and disseminate parts). Following animal slaughter, the specimens were taken quickly and preserved with 10% buffered formalin. Then specimens were dehydrated in an ascending series of ethanol embedded in paraffin wax after its cleanup in xylene. A rotatory microtome was used for cutting 5μm thick tissue sections. Sections were stained using Eosin and Hematoxylin (H&E) [23–24].

For the immunohistochemistry investigation, each animal group was dewaxed in xylene and rehydrated in progressively lower quantities of ethyl alcohol. After that, the sections were properly cleaned in phosphate-buffered saline (PBS). Following that, tissue sections were rehydrated in PBS after being deparaffinized in xylene and cleaned in ethanol and alcohol. For 15 min, antigen retrieval was carried out in a microwave oven using 0.01 M PBS (pH 7.4). The parts were then allowed to cool to room temperature before being cleaned in PBS once again. For thirty minutes, 3% hydrogen peroxide was used to suppress endogenous peroxidase. Goat serum (10%) was used for 20 min after three rounds of washing in PBS to prevent non-specific responses. Next, the polyclonal rabbit anti-AQP5 primary antibody was used (Abcam, Cambridge, UK dilution 1:200) according to the manufacturer’s instructions. The sections were then incubated overnight in a wet chamber. Sections were incubated with biotin-labeled secondary antibodies and avidin-HRP third antibodies; DAB was used to detect the positive staining. Hematoxylin stain was used for section counterstaining. The same procedure applied to negative control sections except for skipping the primary antibody [24].

## Results

### Histologic findings

The ductus deferens had a strongly convoluted shape in Saudi Arabian dromedary camels. Its wall in both reproductive and non-reproductive seasons was composed of mucosa, submucosa, muscularis, and adventitia.

### Ductus deferens structure in non-rutting season

**Figure 1.**
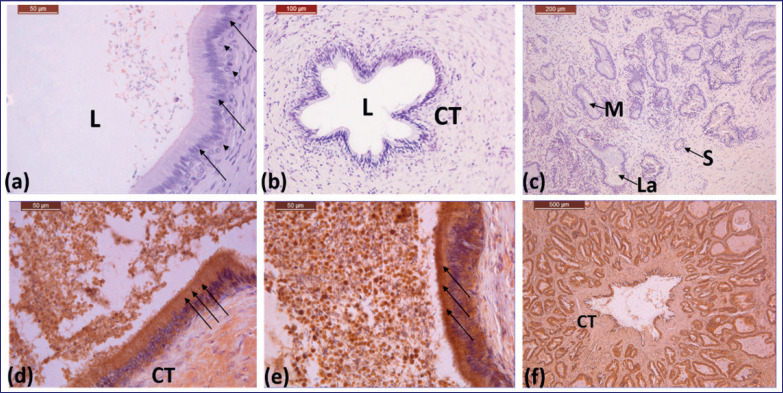
(a)–(c) Photomicrographs showing the initial, middle, and ampulla part parts of ductus deferens of Saudi camel during non-rutting season, respectively: columnar cell (arrow), basal cell (arrow head), connective tissue of submucosal layer (CT), lumen (L). 40X, 20X, 10X. (d)–(f) Photomicrographs showing the immunoreactivity of the initial, middle, and ampulla parts of ductus deferens of Saudi camel during non-rutting season, respectively: lining epithelium (arrows), connective tissue (CT). 40X, 40X, 4X.

In the non-reproductive season, the ductus deferens lining epithelium was pseudostratified, columnar, and covered with cilia (Fig. 1a and b). The epithelium consists of two different cell types: the columnar cell, with a dark oval and basal nucleus, and the basal cell, with a light-colored spherical and central nucleus (Fig. 1a). The lamina propria and the tunica submucosa consist of a thin, loose connective tissue layer (Fig. 1b). Two wide, smooth muscle layers make up the tunica muscularis.

In the non-rutting season, the ductus deferens ampulla gland was tubular-alveolar branched (Fig. 1c). The alveoli of the ductus deferens ampulla were lined with simple or stratified cuboidal epithelium, and spermatozoa and their secretions were found in the lumen. In the non-rutting season, the secretory units had narrow lumina and varied sizes (large, medium, and small) (Fig. 1c). In addition, spermatozoa were seen in the lumen of the ductus deferens in all regions during the non-rutting season (Fig. 1).

### Ductus deferens structure in the rutting season

**Figure 2.**
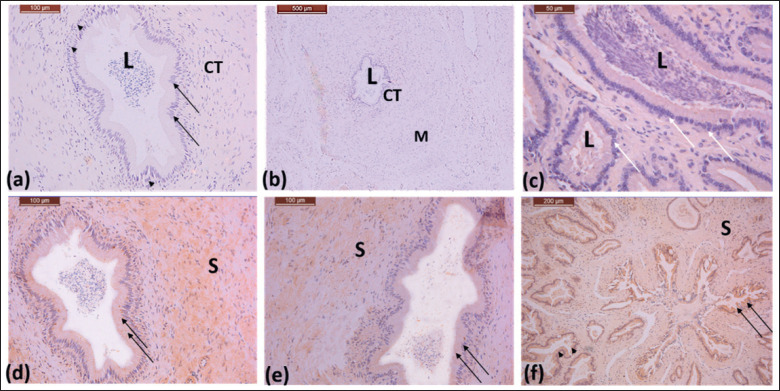
(a)–(c) Photomicrographs showing initial, middle, and ampulla parts of the ductus deference of the Saudi camel during rutting season. The lining epithelium is pseudostratified columnar epithelium: columnar cells (arrow), basal cells (arrow heads), the lumen consists of sperms (L). Connective tissue in the propria submucosa (CT), layers of smooth muscle fibers (M). 40X, 10X, 40X. (d)–(f) Photomicrographs showing immunoreactivity of the initial, middle, and ampulla parts of the ductus deferens of the Saudi dromedary camel, respectively. The lining epithelium of secretory units is stratified cuboidal epithelium (arrows). The lumen consists of a large amount of secretory materials and sperm. Stroma (S). 40 X, 40 X, 20X.

Histologic examination of the ductus deferens during the rutting season revealed that it is lined with pseudostratified columnar epithelium, (Fig. 2a–c). The lumen consists of numerous spermatozoa (Fig. 2a). The ampulla of the ductus deferens appeared as strongly branched alveoli during the rutting season (Fig. 2c). The lining epithelium was shown in Figure 2c as stratified cuboidal epithelium. The lumina was large and filled with sperm and secretions in considerable quantities (Fig. 2c).

### Prostate gland structure in non-rutting season

**Figure 3.**
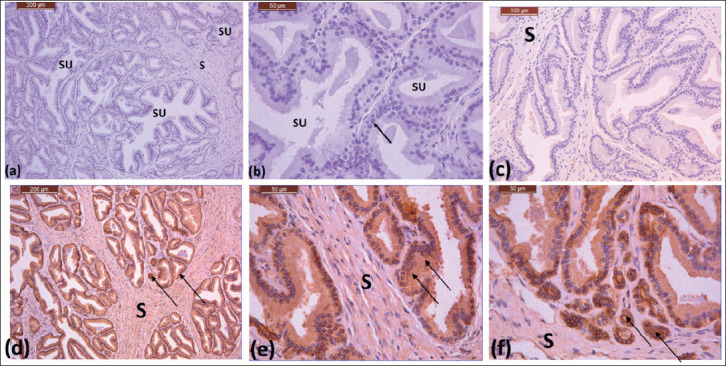
(a)–(c) Photomicrographs showing the body of the prostate gland of the Saudi camel during the non-rutting season, dense stroma (S), secretory units with different shapes and sizes (SU), and a line with stratified cuboidal cells (arrow). 10X, 40X, 40X. (d) and (e) Photomicrographs showing the body of the prostate gland in Saudi camel during non-rutting season. (f) showing the disseminated part. Lining epithelium (arrows), stroma (S). 10X, 40X, 40X,

**Figure 4.**
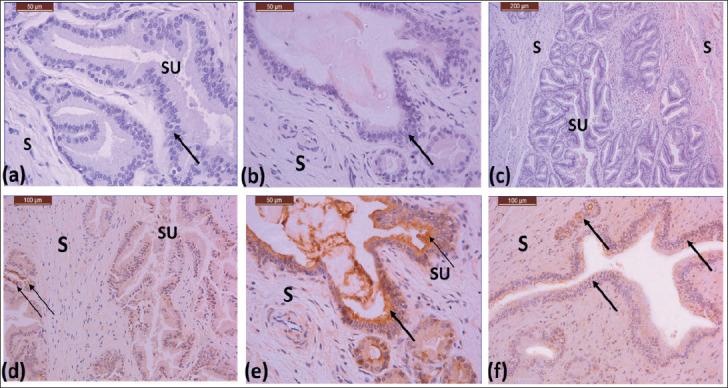
(a)–(c) Photomicrographs showing the body of the prostate gland of the Saudi camel during rutting season, dense stroma (S), secretory units with different shapes and sizes (SU), and a line with stratified cuboidal cells (arrow). 40X, 40X, 10X. (d)–(f) Photomicrographs showing immunoreactivity of the body of the prostate gland in a Saudi camel during rutting season. Lining epithelium showing moderate reaction (arrows), stroma showing mild reaction (S) 20X, 40X, 20X.

In Saudi Arabian dromedary camels, the prostate gland was covered by a thin mesothelium. As in the non-reproductive season, the body of the prostate has a dense stroma of connective tissue and smooth muscle fibers (Fig. 3a and c). The tubuloacinar secretory units that make up the parenchyma have a variety of sizes and shapes, ranging from small to large (Fig. 3a–c). The tubuloacinar secretory units are lined by stratified cuboidal cells (Fig. 3b). The disseminated region of the prostate gland showed less stroma. The parenchyma showed secretory units that were irregular in shape and varied in size from small to large. The lumina of the tubuloacinar secretory units of the prostate gland contain secretion components during both seasons (Figs. 3b and 4b).

### Prostate gland structure in rutting season

The secretory units of the prostate gland in Saudi Arabian dromedary camels during the rutting season have tubuloacinar secretory units with lobular. Stratified cuboidal secretory epithelial cells form the lining epithelium (Fig. 4a–c). Stratified cuboidal cells with dark nuclei were seen in the disseminated region of the prostate gland. With secretion in their lumen, the tubuloacinar secretory unit lumen displayed an irregular shape due to the presence of secretion in their lumen (Fig. 4).

### Immunoreactivity of Aquaporin 5 (AQP-5) in the non-rutting season

The seasonal immunoreactivity of the ductus deference and prostate for AQP-5 during breeding and non-breeding seasons is shown in Tables 1 and 2, and Figures 1–4. Positive AQP-5 immunostaining was found in the initial, middle, and ampulla of the ductus deference in both seasons. The lining epithelium of the initial and middle sections of the vas deferens showed strong reactivity to AQP-5 during the non-rutting season (Fig. 1d and e). The connective tissue and the muscle mantle showed a moderate response (Fig. 1d and f). A strong AQP-5 response was observed in the lining epithelium of the ductus deference ampulla, while mild responses were observed in the muscle sheath and connective tissue (Fig. 1f).

**Table 1.** Seasonal immunoreactivity of AQP5 in the Saudi Arabian dromedary camel ductus deference.

Season	IDD			MDD			ADD			
	LE	MC	CT	LE	MC	CT	LE	AE	MC	CT
Rutting	+	++	+	+	+	++	+++	+++	++	++
Non-rutting	+++	++	+	+++	+	++	++	+++	++	++

Abbreviations: + mild, ++ moderate, +++strong, IDD initial part of ductus deference, MDD Middle part of the ductus deference, ADD ampulla part of the ductus deference, LE lining epithelium, MC muscular coat, CT connective tissue, AE alveolar epithelium.

**Table 2.** Seasonal immunoreactivity of AQP5 in the Saudi Arabian dromedary camel prostate gland.

Season	BP				DP			
	LE	AE	MC	CT	LE	AE	MC	CT
Rutting	+	+	+	+	++	++	+	+
Non-rutting	+++	+++	++	++	+++	+++	++	++

Abbreviations: + mild, ++ moderate, +++strong, BP body of the prostate gland, DP disseminate part of prostate gland, LE lining epithelium, AE alveolar epithelium, MC muscular coat, CT connective tissue.

The disseminated prostate gland, as well as the lining and secretory epithelium of the body, showed a strong granular response to AQP-5 (Fig. 3d–f). The stroma, which is composed of connective tissue and the smooth muscle sheath, showed a moderate response (Fig. 3d–f).

### Immunoreactivity of AQP-5 during the rutting season

Throughout the reproductive season, the lining epithelium of the initial, middle, and ampullary sections of the vas deferens showed a mild to moderate response of AQP-5 (Fig. 2d–f). The muscle sheath showed a moderate response, and connective tissue showed a mild reaction of AQP-5 (Fig. 2d–f). A mild response of AQP-5 was detected in the body of the prostate gland (Fig. 4d–f). During the rutting season, the body and disseminated parts of the prostate gland exhibited mild to moderate AQP-5 reactions.

## Discussion

The main outcomes established by this research were that throughout both breeding and non-breeding seasons, the secretory end pieces of the ampulla of the ductus deferens, both the body and the disseminate sections, have remarkable development. During reproductive and non-reproductive seasons, sperm were seen in the ductus deferens lumen. AQP-5 was discovered in a variety of structures of the initial, middle, and ampulla of the ductus deference and the body and disseminated part of the prostate gland during reproductive and non-reproductive seasons. The reaction was intense in the non-breeding season (summer).

The ductus deferens (initial, middle, and ampulla portions) in rutting and non-rutting seasons were in line with pseudostratified columnar epithelium, according to the study’s results. These findings agreed with [9] for all domestic mammals and [15,16] for dromedary camels. Nonetheless, basal cells in bulls have small lipid droplets [9]. [25] reported that the stratified cuboidal epithelium of the ductus deferens in white roosters. The Al-Ahsa Native Rooster’s vas deferens, however, exhibited a straightforward columnar epithelium [26]. The current investigation contrasted with the latter findings.

According to the current analysis, the ductus deferens lamina propria-submucosa was a thin layer of loose connective tissue in dromedary camels. This result is in agreement with camels [15,16]. The propria-submucosa, or loose connective tissue, is abundant in fibroblasts and elastic fibers and extensively vascularized in other domestic animals and the white rooster [9,25].

According to the current findings, the initial, middle, and ampulla parts of the ductus deferens contained two layers of smooth muscle fibers that constituted the tunica muscularis. This outcome is consistent with research on camels [16].

The current results clarified that the secretory units of the ampulla of ductus deferens showed different histological structures between both reproductive and non-reproductive seasons. The secretory units in the non-rutting season were lined with simple or stratified cuboidal epithelium and had narrow lumina of different sizes. However, the ampulla of the ductus deferens in rutting season appeared as highly branched alveoli, lined with stratified cuboidal epithelium. The present result disagreed with [18]; in the dromedary camel, the ampulla gland is centrally located, narrow, and peripherally located, with wide alveoli bordered with a low columnar or cuboidal epithelium. The majority of the contents of the ampulla gland were secretory materials and spermatozoa.

The results of this experiment demonstrated the highly convoluted anatomy of the ductus deferens in Saudi Arabian dromedary camels. This result supports what [27] found regarding dromedaries and Bactrian camels.

According to a recent study, spermatozoa were found in the lumen of various ductus deferens regions during both rutting and non-rutting seasons. Additional research is required to examine testis immunohistochemistry and acrosome enzymes to investigate the capability of Saudi Arabian dromedary camel fertilization in non-rutting season.The results of this experiment showed that the prostate gland secretory units, which can take on a variety of shapes throughout the non-rutting season, are tubulo-acinar including spherical, oval, and irregular shapes, as well as sizes ranging from small to large. Stratified cuboidal cells lined the tubuloacinar units. In rutting season, secretory units had a highly lobulated arrangement. Stratified cuboidal epithelium lines the prostate gland’s body and disseminates portions. These results disagreed with [17], who stated that the parenchyma of the prostate gland during the rutting season formed compound tubulo-alveolar units. The alveoli and tubules were lined by high columnar cells with few basal cells.

According to [28], uncastrated bulls had thick fibrous capsules covering their prostates that were made of coarse collagen and sparse elastic and smooth muscle fibers. Tall columnar epithelium lined the alveoli. The present investigation disagreed with the later author.

The findings of the current investigation showed that the sperm were observed in the lumen of the ductus deferens of the Saudi Arabian dromedary camel in both reproductive and non-reproductive seasons. The results agreed with the finding of [5], who clarified that male camels are known to reproduce only during certain seasons, reaching their sexual peak (the rut) during the mating season and going through the rest of the year without having any sexual activity. However, the male camel can still mate and fertilize a female in estrus at any time of the year.

Several researchers found that the aquaporin in animals [29,30,31,32]. The results of this study, which examined AQP-5 in Saudi Arabian dromedary camels for the first time, demonstrated that the lining epithelium of the initial, middle, and ampulla parts of the ductus deferential displayed a strong immune reactivity to AQP-5 during the non-rutting season. Additionally, there was a high granular reaction of AQP5 in the prostate gland’s disseminated section as well as the lining and secretory epithelium of the body. During the rutting season, mild to moderate AQP-5 reactions were observed in the acinar and lining epithelium of the initial, middle, and ampulla sections of the vas deferens. The body and a disseminated portion of the prostate gland exhibited a mild to moderate response to AQP-5. The current finding conflicts with [31], who claimed that the prostate gland of rutting camel males had higher immunological reactivity levels of AQP-7 than the ductus deferens.

## Conclusions

This study may help highlight the expected function of AQP-5 in male camels during both reproductive and non-reproductive seasons. Consequently, during both breeding and non-breeding seasons, determining the immunologic reactivity levels of the anti-AQP-5 antibody, a measure for energy and water balance, is critical in the ductus deferens and prostate gland in male camels. In both reproductive and non-reproductive seasons, the initial, middle, and ampullary sections of the ductus deferens were lined with pseudostratified columnar cilia. Cuboidal epithelium, either simple or stratified, lined the ampulla secretory units. Secretory components were present in the lumen of secretory units during both the non-rutting and the rutting seasons. Throughout the breeding and non-breeding seasons, sperm were found in the lumen of the ductus deferens. The epithelial cells of the prostate gland and vas deferens may also be more dependent on AQP-5 during non-rutting seasons than during rutting seasons. According to the study’s conclusions, Saudi Arabian dromedary camels are capable of breeding both during and outside the rutting season.

## List of Abbreviations

ADD, ampullary part of the ductus deference; AE, Alveolar epithelium; AQP0, Aquaporin 0; AQP-5, Aquaporin5; AQP12, Aquaporin12; AQPs, Aquaporins; BP, body of the prostate gland; CT, connective tissue; DP, disseminate part of prostate gland; H&E, Hematoxylin and Eosin; IDD, initial part of ductus deference; L, lumen; LE, lining epithelium; M, smooth muscle fibers; MC, muscular coat; MDD, Middle part of the ductus deference; PBS, phosphate buffered saline; S, stroma; SU, secretory units.
